# The impact of school closure and social isolation on children in vulnerable families during COVID-19: a focus on children’s reactions

**DOI:** 10.1007/s00787-021-01758-x

**Published:** 2021-03-26

**Authors:** Linda Larsen, Maren Sand Helland, Tonje Holt

**Affiliations:** Division of Mental and Physical Health, Norwegian Institute of Public Health, Skøyen, P.O. Box 777, 0213 Oslo, Norway

**Keywords:** Social isolation, Home school, COVID-19, Child reactions, COVID-19 related predictors

## Abstract

For children the consequences of the COVID-19 public health measures may have long-term effects into adulthood. By exploring children’s reactions more broadly, we are better placed to understanding the breadth of implications of home school and social isolation under COVID-19. The present study explored how COVID-19 related variables, namely, home school experience, child perceived family stress and instability, screen time use, missing friends and worry about virus infection are associated with children’s emotional, somatic/cognitive and worry reactions, respectively. A total of 442 children (*M* = 11.43 years, SD = 2.59) from the longitudinal FamilieForSK-study participated and a series of hierarchical linear regression models were applied controlling for background variables including children’s psychological vulnerability. Results showed significant associations between all COVID-19 related predictors, except screen time use, and the three outcomes. Family stress and instability had the strongest effects with standardised betas ranging from .356 to .555 and collectively, predictors explained between 20.7 and 44.1% of variance in outcomes. Furthermore, several associations were moderated by age and older children were more negatively impacted (i.e., higher level of reported reactions). The present study provides more conclusive evidence of the effects of home school and social isolation under COVID-19 on children. It also exemplifies the importance of focusing on children’s reactions more broadly, as there was evidence that children on average had fewer emotional reactions compared to before the pandemic.

## Introduction

On March 11, 2020, the World Health Organisation (WHO) called on countries to take urgent and decisive action against the coronavirus disease (COVID-19) [1]. Governments around the world introduced strict public health measures to mitigate the spread of the disease, and while these measures have indeed been effective [2, 3], there are indications of deleterious mental health effects on children and adults [4–7]. It is particularly important to examine the effects on children, not only as research is scarce [8], but also as children are vulnerable to environmental changes and effects may have long-term consequences into adulthood [9]. We propose that effects on children should be studies more holistically by exploring a broader range of reactions rather than focusing on symptomatology of mental health difficulties. To this end, the present study focuses on a broader spectrum of children’s emotional reactions, along with children’s somatic and cognitive reactions, and worry reactions relating to parents and family. By exploring the normal range of reactions, we are better placed to understanding the breadth of implications of the pandemic on children.

## Consequences of school closure and social isolation under COVID-19

The biggest change to children’s daily lives under COVID-19, might have been the closure of schools and introduction of digital home schooling. School is a place of academic learning, but also an arena for development, socialization and connecting with friends and peers, and for emotional and academic support from teachers, which are all important factors for children’s psychological wellbeing and adjustment [10–13]. School routines further allow children to have regular bed/wake times and physical activity, and restricts sedentary behaviours and/or non-educational screen time [14]. Conversely, school closures under COVID-19 has been associated with academic learning losses [15] and an array of health risk behaviours (e.g., socio-emotional complications, reduced physical activity) [16].

A marked difference between school closure under COVID-19 and more regular school closure (e.g., during holidays, teacher strikes), is that students continued their schoolwork digitally in the absence of physical contact. Children have varied in their experience with the new school day, in terms of among other things, their ability to concentrate on school work, meeting assignment deadlines, and their perceived level of support (or lack thereof) from teachers and parents. For some it may have been a blessing with increased independence, while for others, it would have been a struggle with motivation and self-discipline. And in particular, younger children seem to have done more poorly [17, 18].

Home schooling under COVID-19 was accompanied by strict social isolation measures and thus, children had involuntary restrictions placed on their opportunities to meet friends and peers physically although they may not have adhered (strictly) to these recommendations [19]. Evidence suggests that social isolation during the pandemic was associated with loneliness, negative consequences on mental health and other health-related behaviours for children [20, 21]. For example, two Chinese studies report elevated levels of symptoms of depression and anxiety in children and adolescents during the early phase of the pandemic. Children who worried about virus infection were significantly more likely to experience symptoms of depression, but not anxiety, compared to those that were not or only slightly worried about being infected [5, 22]. Furthermore, research from North American has found that COVID-19-related worry and stress along with digital time spent with friends was associated with more loneliness and depression in adolescents, while time spent doing homework was negatively associated with depression and, therefore, acted as a buffer [23]. From a Scandinavian and European context, a Norwegian study has shown that loneliness and mental health problems in adolescents were associated with social media use and a lack of physical contact with friends [24], while a Spanish study has shown that home confinement was associated with reduced physical activity and increased screen time and sleep time [21].

These studies point towards some of the risk and (to a lesser extent) protective factors for children and adolescents during social isolation. However, it is possible that the results are not so much about the COVID-19 situation, as it is about having difficulties before and continuing through the pandemic, and that the same factors are associated with these difficulties. To provide stronger empirical evidence, we need longitudinal rather than cross-sectional studies, where it is possible to control for any pre-existing psychological vulnerabilities.

## Consequences for the family

The division of labour and daily routines in families changed dramatically during the COVID-19 lockdown [25], something that may dampen or amplify the effects of school closure on children. Such family disruptions may broadly be described as affecting the family’s routines, rituals and rules [26], which collectively, these fall under the umbrella term of organizational processes according to Walsh’s family resilience framework [27]. The sudden changes to family routines included, for example, the reallocation of household tasks, children and parents spending more time at home due to home school and home office/parent being furloughed, and moving between homes for children of divorced parents. Changes to rituals include religious or cultural celebrations, and lastly, rule changes may include parents renegotiating rules for when/if children are able to leave the home or new rules for school in the context of home-schooling [26]. Unsurprisingly, these disruptions have led to increased household tensions [25] and there are reports of increased interparental conflicts [28], while others report that taking care of children was rated as a positive experience by parents [29]. As the pandemic-related consequences felt by parents may cascade down to children, we might expect to see this reflected in children’s reactions and wellbeing [26]. Studies are yet to explore how children’s positive and negative reactions to the COVID-19 pandemic relate to concurrent and former family functioning.

## Disproportionate emphasis on negative consequences

There has been a disproportionate emphasis on the negative consequences of the measures introduced to mitigate the spread of the pandemic. However, some argue that the consequences might not be all negative and in fact, some children and families have fared better [30]. For example, the lockdown measures may have reduced daily stressors for some children and families, and the sudden increase in family time may have been another positive for some. We agree with Bruning and colleagues [30] in the importance of taking a more holistic approach focusing on the heterogeneity of experiences under COVID-19 and thereby, in our attempt at understanding the impact on children shifting the focus from symptoms of psychopathology and mental health difficulties to the normal range of reactions. This may be achieved by asking more moderate questions like “I felt sad” and “I have had trouble sleeping at night”, rather than “I felt miserable or unhappy” and “I didn’t enjoy anything at all”, which have a much stronger affective phrasing. Coupled with a longitudinal study design, we are better placed to providing a more reliable and nuanced picture of the effects of the COVID-19 public health measures on children including any direct effects by exploring children’s reactions or wellbeing prior to and during the lockdown period [31].

## Consequences for children of different ages

It is noteworthy, that generally and under COVID-19, the impact of social isolation (and loneliness) on the mental health of children has been disproportionately explored in adolescent and older children [e.g., 5, 20, 23, 24]. Taking a child developmental perspective, the association between isolation and mental health should be stronger in adolescent and older children [32], given their heightened experience of emotional reactions, underdeveloped self-regulatory mechanisms and heightened motivation for peer affiliation and support. Developmentally, it is a time characterised by increasing independence from parents, increasing autonomy and peer friendships that become closer and more supportive [33]. Thus, there is a gradual shift in focus from the family to peers and friends [34]. The social isolation measures under COVID-19, may have been particularly challenging for older children as these have impeded close physical contact with friends and support from friends. The renegotiation of family rules by parents as mentioned earlier, may also have challenged older children’s developmental trajectory towards independence and autonomy. Therefore, to better understand the implications of home schooling and social isolation on children′s reactions, it is paramount to include a broader age-range of children and to explore the moderating effect of children’s age (as a proxy for their developmental stage) on any observed relationships.

## The present study

Little is about how children have reacted to the new everyday life under COVID-19 with home schooling and social isolation. The available evidence from cross-sectional studies suggests that elevated levels of depression and anxiety during lockdown are related to variables such as worry about virus infection and social media use. Claims that other variables such as home school experiences and family functioning are related to how children react need a better empirical foundation. Longitudinal studies focusing on younger and older children’s reactions more broadly and accounting for any pre-existing psychological vulnerabilities better capture the dynamics of how children have reacted under the COVID-19 pandemic. To this end, we use data from a longitudinal study to explore the following questions:

1. How do children compare their reactions under COVID-19 with home schooling and social isolation to before the lockdown?
2. How are children’s reactions under COVID-19 associated with home school experience, perceived stress and instability in the family, screen time use, missing friends, and worry about virus infection?
3. Does children’s age moderate any of these associations?

## Methods

### Study design and participants

The data for this study were drawn from the Norwegian Family Dynamics Study (FamilieForSK), a longitudinal study aimed at increasing knowledge about family dynamics and conflicts in Norwegian families. The FamilieForSK-study has more than 2300 participating families, recruited through family counselling centres from December 2017 to July 2019 when families attended mandatory mediation (in relation to divorce/relationship dissolution), counselling or family therapy. Participating parents and children completed online questionnaires covering a wide range of topics, while trained interviewers interviewed younger children (7–11 years of age). In some families only one or both parents participated, while in other families the parent(s) and their child(ren) participated and finally, in a small number of families only the child(ren) participated. Shortly after the Norwegian government introduced public lockdown measures to mitigate the spread of COVID-19, FamilieForSK initiated an extraordinary data collection (Wave 3) to explore the experience of these measures on families. Parents and children that had already participated in Waves 1 and 2 were invited to participate in Wave 3, while parents and children due to participate in Wave 2 were invited to participate in Waves 2 and 3 at the same time (i.e., Wave 3 survey was joined with Wave 2 survey). The present study uses data from the children who participated at Wave 3 (April 1 to May 25, 2020), of whom nearly 85% also participated at Wave 1. There were no significant differences between children who only participated in Wave 1(n = 573) and children who participated in both Waves 1 and 3 (*n* = 374) in terms of symptoms of anxiety and depression and whether their parents lived together or lived apart. However, children who participated in Waves 1 and 3 were significantly younger than children who only participated in Wave 1 (*M* = 10.15 (*SD* = 2.53) vs. *M* = 10.91 (*SD* = 2.50), *t*(934) = 4.54, *p* < 0.001). See Table 1 for an overview of the sample characteristics.

**Table 1:** Sample descriptive statistics (*N* = 442)

	%	*n*
Age (*M*, SD)	11.43	2.59
Gender (girls)	55	244
Symptoms of depression (*M*, SD)	0.36	0.34
Symptoms of anxiety (*M*, SD)	0.28	0.30
Family structure		
Child’s parents live together	32.64	141
Child’s parents live apart	67.36	291
Custody arrangement under the pandemic (*n* = 283)		
Live with both parents equally	47.4	134
Live mostly with mum	29.0	82
Live mostly with dad	6.7	19
Live just with mum	14.8	42
Live just with dad	2.1	6
Custody arrangement pre-pandemic (*n* = 286)		
Live with both parents equally	59.1	169
Live mostly with mum	27.3	78
Live mostly with dad	4.2	12
Live just with mum	8.7	25
Live just with dad	0.7	2
Virus infection		
Child been infected	0.45	2
Family member been infected	2.26	10
Quarantine during the pandemic		
Child been in quarantine	10.41	46
Family member been in quarantine	31.67	140
Hospitalisation due to virus infection		
Family member been hospitalised	0.90	4

Symptoms of depression was assessed with the Short Mood and Feelings Questionnaire (MFQ) Short Form and symptoms of anxiety was assessed with five items from the Screen for Child Anxiety Related Disorders (SCARED)

In Norway, the Government closed all schools on March 12 and digital home schooling was introduced. Schools re-opened gradually from April 27 and younger children (up to grade 4) were the first to return to school. The school day was somewhat different to before the pandemic, as class sizes were reduced and children were grouped into smaller cohorts to reduce the possibility of spread of the disease [35]. The present study includes a small number of children who participated after the schools had opened, but note that only four of these children were younger and had potentially returned to school. Furthermore, the majority of children that participated after April 27 did so within just a few days. We decided nonetheless to include all children that had responded up until the point of May 25.

The Regional Committee for Medical and Health Research Ethics in Norway approved the study and all study procedures fulfilled the recommendations of the Helsinki Declaration. Parents consented for children to participate in the FamilieForSK- study and children assented before completing the online survey or before being interviewed by trained interviewers.

### Measures

We assessed children’s reactions to the new everyday life with home schooling and social isolation with ten statements with the item stem “After the schools closed in March, I have …”. Items were developed specifically for the study. An initial Principal Component Analysis (PCA) with a promax rotation showed that items formed three components; Emotional Reactions with five items (e.g., “felt sad”, “felt angry”, “felt lonely”), Somatic/cognitive Reactions with three items (e.g., “had trouble concentrating”, “had headaches, stomach ache and so on”), and Worry Reactions with two items (e.g., “worried about my parents”). Children answered items on a scale from 0 (“A lot less than before”) to 4 (“A lot more than before”), with the middle value (2) representing “As before”. The Emotional and Somatic/cognitive reactions scales had acceptable internal reliability (*α* = 0.79 and *α* = 0.66, respectively) and the two worry items were moderately correlated (*r*_polychoric_ = 0.59, *p* < 0.05). See Tables 3 for descriptive statistics including item factor loadings.

Home school experience was assessed with four statements about how children managed home schooling, their concentration level, and home school support or lack thereof (reverse scored). Items were answered on a scale from 0 (“Not true”) to 2 (“Certainly true”) and internal reliability was acceptable (*α* = 0.72).

Family stress and instability was assessed with three statements tapping children’s perception of parent stress levels, and instability and arguments in the family under COVID-19 restrictions. Questions were answered on a scale from 0 (“A lot less than before”) to 4 (“A lot more than before”) and internal reliability was acceptable (*α* = 0.78).

Daily screen time use (including gaming and social media use, but not school work) was assessed with a single question, “After the schools closed in March, how many hours of screen time have you had each day?” with the response options “0–1 h”, “2–3 h”, “4–5 h” and “more than 5 h”.

Missing friends and worry about virus infection were assess with two statements, namely, “After the school closed in March, I have missed seeing my friends” and “After the schools closed in March, I have been worried about being infected with the coronavirus or infecting others with the coronavirus”. Items were answered on a scale from 0 (“Not true”) to 2 (“Certainly true”).

As an index of children’s psychological vulnerability, we used their scores on the Short Mood and Feelings Questionnaire (SMFQ) [36] and five items from the Screen for Child Anxiety Related Disorders (SCARED) [37] when they first participated in the study (around 18 months earlier). The SMFQ is a 13-item self-report measure of depressive symptoms over the past 2 weeks, while SCARED is a self-report measure of anxiety symptoms over the past 3 months. Both scales have demonstrated good psychometric properties [36–39] and in the present study, internal reliability was 0.85 and.51 for SMFQ and SCARED, respectively. See Tables 2 and 3 for an overview of the primary variables in the study.

**Table 2:** Descriptive statistics and correlations among primary variables

	*M*	*SD*	Range	1	2	3	4	5	6	7	8	9
1. Emotional reactions	1.89	0.65	0–4									
2. Somatic/cognitive reactions	2.10	0.66	0–4	0.57								
3. Worry reactions	2.00	0.74	0–4	0.49	0.44							
4. Home school experiences	1.56	0.45	0–2	− 0.33	− 0.41	− 0.14						
5. Family stress and instability	1.95	0.63	0–3.40	0.63	0.46	0.41	− 0.24					
6. Screen time usage^a^			0–3	0.01	0.11	− 0.04	− 0.27	− 0.03				
0–1 h	56	12.90										
2–3 h	144	33.18										
4–5 h	108	24.88										
> 5 h	126	29.03										
7. Missing friends^a^			0–2	0.16	0.10	0.08	− 0.06	0.16	− 0.06			
Not true	18	4.10										
Somewhat true	77	17.54										
Certainly true	344	78.36										
8. Worry virus infection^a^			0–2	0.17	0.15	0.14	− 0.15	0.07	0.18	0.25		
Not true	225	51.25										
Somewhat true	168	38.27										
Certainly true	46	10.48										
9. Symptoms of depression	0.36	0.34	0–1.77	0.19	0.27	0.10	− 0.31	0.20	0.16	0.12	0.12	
10. Symptoms of anxiety	0.28	0.36	0–1.60	0.18	0.12	0.11	− 0.13	0.11	0.03	0.08	0.08	0.49

*M* Mean. *SD* Standard deviation

Pearson correlations are reported between numeric variables, polychoric correlations between categorical variables, and polyserial correlations between categorical and numeric variables

^a^Descriptive statistics are reported as frequencies (*N*) and percentages (%)

**Table 3:** Descriptive statistics for individual reaction items included in children’s reaction dimensions

Subscales	Questionnaire items	*N*	Factor loadings	*M*	*SD*	Range	*t*-test statistic
	After the schools closed in March, I have …						
Emotional reactions	… felt sad	441	.86	1.81	0.87	0–4	*p* < .001
	… felt scared or uneasy	438	.60	1.88	0.84	0–4	*p* < .01
	… felt angry	435	.66	1.90	0.82	0–4	*p* < .01
	… felt unsafe	428	.66	1.72	0.82	0–4	*p* < .001
	… felt lonely	438	.58	2.14	1.01	0–4	*p* < .01
Somatic/cognitive reactions	… had trouble concentrating	438	.67	2.17	0.93	0–4	*p* = .001
	… had headaches, stomach ache and so on	434	.53	2.02	0.84	0–4	*p* = .69
	… had trouble sleeping or sleeping through the night	439	.78	2.10	0.82	0–4	*p* < .05
Worry reactions	… been worried about my parents	439	.85	2.00	0.79	0–4	*p* = .952
	… been worried about my family’s future	433	.78	2.00	0.88	0–4	*p* = .957

Children answered the reaction items on a scale from 0 (“A lot less than before”) to 4 (“A lot more than before”)

Test statistics testing if responses are significantly different to before COVID-19 (i.e., score = 2 (“As before”))

### Analytic strategy

Data analyses were performed in R [40] using the mice [41], sjPlot [42], polycor [43], miceadds [44] and psych [45] packages. We performed initial item and scale inspections by calculated descriptive statistics and bivariate correlations. We then used a series of *t*-tests to explore if children’ rated their reactions (at the item level and scale level) as significantly different to before. This was achieved by specifying the mu argument to be equal to two (i.e., the value of the response option corresponding to “As before”). Prior to the main analyses, we imputed missing data on predictors, covariates and outcomes using multiple imputation by chained equations [41]. The percentage of missing data was generally low (< 3%), with the exception of MFQ and SCARED, where missingness was 17%. Seventy-three to 74% of the 442 children would have been available for the main analyses under the traditional listwise deletion method. Five imputed datasets were generated and regression analyses run on each dataset was pooled according to Rubin’s rules [46]. Regression results did not differ when we used incomplete data with listwise deletion or complete cases only, and thus, to utilise all available data we present the results from imputed data. In the main analyses, we performed a series of hierarchical linear regressions with Emotional Reactions, Somatic/cognitive Reactions and Worry Reactions as outcome, respectively, and background variables entered in Step 1, and COVID-19 predictor variables entered in Step 2. Finally, we explored age interactions in a series of regression models, where background variables and COVID-19 predictors were entered in Step 1 and each age interaction entered in Step 2.

## Results

### Descriptive and preliminary analyses

Table 2 provides means, standard deviations, frequencies and correlations for the primary variables in the study. Unsurprising, the majority of children (78%) reported that they missed their friends and 51.25% worried about being infected or infecting others with the coronavirus. Over half the children reported that their daily screen time usage was four hours or more. Using *t*-tests we explored if children’s reactions were significantly different to before (i.e., a test value of 2 = “As before”) and found that this was indeed the case for most items, with the exception of “had headache, stomach ache and so on”, “worried about my parents” and “worried about my family’s future” (see Table 3). This suggests that children felt less sad, scared/uneasy, angry and unsafe (as mean scores were < 2), but more lonely and had more difficulty concentrating and sleeping at night (as mean scores were > 2) compared to before the government initiated the schools closures. Furthermore, at the scale level, we observed significant results for Emotional Reactions and Somatic/cognitive Reactions, *t*(440) = -3.57, *p* < 0.001 and *t*(440) = 3.01, *p* < 0.01, respectively. As the average for Emotional Reactions was 1.89, this means that children on average reported fewer emotional reactions compared to before. For Somatic/cognitive Reactions the average was 2.10 and thus, children on average reported more somatic/cognitive reactions compared to before (see Table 2).

### Main regression analyses

To explore how children’s Emotional, Somatic/cognitive and Worry reactions, respectively, are associated their experiences and living situation during the pandemic, three hierarchical regression models were estimated (one for each outcome). In the first two models, we found that home school experience and perceived stress and instability in the family during COVID-19 restrictions were associated with children’s Emotional and Somatic/cognitive Reactions, respectively, after controlling for background variables and other predictors in the models. Children who had a more positive home school experiences reported lower Emotional and Somatic/cognitive Reactions, respectively, while children who experienced higher levels of stress and instability in the family reported more Emotional and Somatic/cognitive Reactions. Increased perceived stress and instability in the family was also associated with more Worry Reactions. Furthermore, we found that compared to children who did not miss their friends, those that did miss their friends reported significantly higher Emotional Reactions on average. Finally, compared to children who said they were not worried about virus infection, those that said they were certainly worried about virus infection reported significantly higher Emotional and Worry Reactions, respectively. The three models, respectively, accounted for 46, 33 and 23% of the explained variance in Emotional, Somatic/cognitive and Worry Reactions during lockdown. See Table 4 for an overview of the results. Results were similar when we used the initial dataset with listwise deletion or complete cases only, exception for missing friends, which was non-significant possibly due to a lack of statistical power.

**Table 4:** Summary of hierarchical regression analyses for variables predicting child reactions (*N* = 442)

Predictors	Emotional reactions				Somatic/cognitive reactions				Worry reactions			
	*B*	*SE B*	*β*	*p*	*B*	*SE B*	*β*	*p*	*B*	*SE B*	*β*	*p*
Control variables												
(Intercept)	1.263	0.221	− 0.492	*p* < 0.001	1.964	0.254	− 0.210	*p* < 0.001	1.842	0.307	0.174	*p* < 0.001
Gender	0.048	0.053	0.075	0.367	0.148	0.060	0.223	*p* < 0.05	− 0.012	0.069	− 0.017	0.857
Age	− 0.034	0.011	− 0.136	*p* < 0.01	− 0.018	0.012	− 0.068	0.155	− 0.048	0.015	− 0.171	*p* < 0.01
Symptoms of depression	− 0.046	0.087	− 0.025	0.595	0.185	0.102	0.096	0.072	− 0.068	0.115	− 0.032	0.556
Symptoms of anxiety	0.140	0.107	0.065	0.198	− 0.142	0.115	− 0.064	0.220	0.051	0.129	0.021	0.694
Interest variables												
Home school experiences	− 0.295	0.059	− 0.204	*p* < 0.001	− 0.425	0.067	− 0.287	*p* < 0.001	− 0.107	0.080	− 0.066	0.179
Family stress and instability	0.569	0.040	0.555	*p* < 0.001	0.375	0.045	0.356	*p* < 0.001	0.485	0.055	0.417	*p* < 0.001
Screen time usage	0.007	0.026	0.010	0.800	0.032	0.030	0.050	0.283	0.026	0.037	0.036	0.494
Missing friends												
Somewhat true	0.266	0.129	0.412	0.040	− 0.062	0.145	− 0.094	0.669	− 0.271	0.175	− 0.369	0.123
Certainly true	0.262	0.119	0.405	0.029	0.047	0.135	0.071	0.726	− 0.149	0.163	− 0.203	0.363
Worry virus infection												
Somewhat true	0.026	0.051	0.041	0.609	0.068	0.058	0.102	0.245	0.037	0.069	0.050	0.595
Certainly true	0.285	0.085	0.441	*p* < 0.01	0.055	0.093	0.082	0.558	0.278	0.113	0.379	*p* < 0.05
*R*^2^/*R*^2^ adjusted	0.455/0.441				0.327/0.309				0.227/0.207			
*F* for change in *R*^2^	45.107***				21.855***				15.334***			

Mean scores were used for outcomes, background variables and numeric predictors, while dummy variables were used for Missing friends and Worry about virus infection with “Not True” as the reference category (coded 0). Boys were the reference group (coded 0) for gender

All regression coefficients are from the second step in the analyses and *F* change statistics are calculated using multivariate Wald statistic from the mice package

***p < .001

Finally, from our exploration of age as a moderator of the relationship between COVID-19 related predictors and children’s reactions, we found several significant interaction effects after controlling for the main effects of the covariates and other predictors (see Table 5). First, age moderated the association between family stress and all three reaction dimensions. Thus, the older children were the more negatively impacted they were by family stress and instability in terms of their reactions to the new everyday life under COVID-19. Age also moderated the association between screen time use and Somatic/cognitive Reactions with a stronger association for older compared with younger children. Furthermore, we found a significant age by missing friend’s interaction for Somatic/cognitive Reactions, while for Worry Reactions the effects was marginally significant. Thus, the association between age and missing friends was generally stronger for older children when one compared those that missed their friends most to those that did not miss their friends. Finally, we found a significant age by worry about virus infection interaction for Somatic/cognitive Reactions and Worry Reactions. This suggests that for older children, the magnitude of the effect of worry about virus infection on Somatic/cognitive and Worry Reactions is stronger when one compared children who were somewhat worried about virus infection to those that were not worried about virus infections.

**Table 5:** Summary of hierarchical regression interaction analyses for variables predicting child reactions (*N* = 442)

Interactions	Emotional reactions				Somatic/cognitive reactions				Worry reactions			
	*B*	*SE B*	*β*	*P*	*B*	*SE B*	*β*	*p*	*B*	*SE B*	*β*	*p*
Family stress × Age	0.053	0.015	0.086	*p* < .001	0.051	0.017	0.083	*p* < .01	0.073	0.020	0.118	*p* < .01
Screen time usage × Age					0.027	0.011	0.071	*p* < .05				
Missing friends												
Certainly True × Age					0.138	0.059	0.359	*p* < .05	0.142	0.073	0.368	*p* = *.05*
Worry about virus infection												
Somewhat True × Age					0.047	0.023	0.121	*p* < .05	0.083	0.027	0.214	*p* < .01

For ease of presentation interaction effects are presented together for each outcome, although we note that each interaction was tested in a separate model adjusted for background variables and all other predictors (i.e., models akin to those reported in Table 4). Only significant interaction effects are shown

## Discussion

In this study, we use data from a longitudinal study to shed light on children’s reactions to the new everyday life under COVID-19 with home school and social isolation. We focused on children’s reactions more broadly, as this allowed us to investigate the normal range of reactions and gives a more nuanced view on the implications of the pandemic for children. Specifically, we explored children’s Emotional, Somatic/cognitive and Worry Reactions, and how each of these reaction dimensions relate to variables expected to be pertinent to change under the COVID-19 restrictions.

We asked children how they compared their reactions under COVID-19 with home school and social isolation to before the schools were closed and found that children reported fewer emotional reactions, but more somatic/cognitive reactions. That is, children on average coped better emotionally as they felt less sad, scared, angry and unsafe, but did more poorly in terms of sleep and concentration. That children should experience fewer emotional reactions is in line with certain findings from adults showing only mild levels and no increases in anxiety and depression symptomology [47, 48]. While our results contrast with studies showing increased levels of anxiety and depression in children and adolescents [5, 22], it does, however, provide empirical support for the notion that child wellbeing might be improved under COVID-19 [30]. One possible explanation for this is that children received more attention and support than usual from their parents as families spent more time together. Alternatively, school closure, albeit with the introduction of digital home schooling, might have provided children with a break or respite from school-related worries and pressures (e.g., keeping up with others, complex social relations). As to why children reported significantly more somatic/cognitive reactions compared to before, this may relate to the changes in daily routines for children such as a shift in bed/wake times or less physical activity [14, 16]. Children’s worry reactions pertaining to their parents and family were unchanged relative to before, which we tentatively see as positive. Although, we cannot rule out that children had a high level of concern for their family and parents prior and maintained this during the pandemic, as we did not ask children to rate their level of worry per se.

Almost all of the COVID-19 related predictors were associated with children reactions during the pandemic. The strongest predictor of child reactions was children’s perceived family stress and instability, which was significantly and positively associated with all three child-reaction dimensions. Given the interrelatedness of family subsystems [49] and evidenced relationship between child adjustment and general family climate [50], the effects of parent stress and family instability on children under COVID-19 is not surprising. Following Prime and colleagues [26], the effects might have cascading through to children resulting in them reporting more negative reactions (i.e., higher reaction scores) for all three dimensions. We emphasize that in the present study, we controlled for children’s pre-pandemic psychological wellbeing and thus, we have been able to “eliminate” the effect family stress and difficulties usually have on children’s wellbeing. Interestingly, we note that children on average rated the level of family stress and instability to be similar to before the pandemic using our retrospective measure. Why might this be, if families experienced substantial disruptions particularly to family routines and rules? The gravity and extraordinary nature of the COVID-19 situation might somehow have enabled families to mobilize resources to protect the family and its subsystems in the face of pandemic-related stressors [51, 52] or while purely speculative, families might have felt some “comfort” in knowing that they were not alone in their experiences.

Screen time was the only predictor that was not significantly associated with any of the reaction-dimensions. At least one study has found that social media use, but not online gaming, is predictive of loneliness and symptoms of anxiety and depression in youths during pandemic lockdown [20]. We do not know what type of activities children in our study were doing on screens. This, along with study differences in terms of screen time measures and samples, and the fact that we partialled out the effect of pre-pandemic psychological vulnerability might collectively explain the divergent results. During the pandemic, meeting friends digitally was for many children the only opportunity to meet, and thus, the association between screen time and reactions might have been weaker during the pandemic relative to the “normal situation”, where children and adolescents can still meet physically.

We hypothesized that possibly the biggest change for children was the closure of schools and introduction of digital home school. Our results indicate that positive home school experiences acted as a buffer on children’s reactions to the new everyday life; children who did better with home schooling reported fewer emotional and somatic/cognitive reactions. Home schooling likely gave children more autonomy over their day, and it would have required a high level of self-discipline and motivation to establish good routines and succeed with home schooling. One might wonder if children who succeeded at this, were generally well-adjusted and this reflected in their reported reactions. However, our results counter this argument as we controlled for children’s prior psychological wellbeing (or vulnerability), and thus, the observed effect is above and beyond any effect of children’s general adjustment. If a more positive experience of home school has the potential to buffer children against more negative reactions, then school administrations and teachers must ensure that home schooling during a crisis-situation like COVID-19 is optimally put together and delivered to meets students’ needs including teacher support.

Naturally, many children missed their friends and our results indicate that children who missed their friends also reported significantly higher levels of emotional reactions than children who did not miss their friends. Friendships play a large role in children’s lives, not least in terms of a means of support, and our result might indicate that children’s need for emotional support from friends was not met due to the social isolation measures and the absence of usual contact at school, and therefore, they experience heightened emotional reactions.

Finally, our results indicate that children who worried about virus infection reported more emotional and worry Reactions, respectively, compared to children who were not worried about virus infection. This could not be attributed to children’s general emotional vulnerability as we controlled for this in the analyses. Thus, children’s concerns about the coronavirus seem to have direct implications for their emotional response and worries for their family and parents. The association with children’s emotional reactions is particularly important as it demonstrates, for the first time that worry about virus infection is associated with a broader spectre of emotional reactions and not just symptoms of mental health difficulties [5, 22].

Taking a child developmental perspective, we explored if children’s age moderated any of the relationships between COVID-19 predictors and children’s reactions. We did indeed find that age moderated the relationship between family stress and instability and all three reaction-dimensions. Age also moderated the relationship between screen time and somatic/cognitive reactions, and the relationship between missing friends and worry about virus infection and somatic/cognitive and worry reactions, respectively. All significant coefficients were positive, suggesting that relationships were stronger for older children compared to younger children. That family stress and instability is more strongly associated with emotional reactions in older children fits with the developmental perspective of adolescence as a time of sensitivity and of heightened emotional reactions [34]. Another possibility is that older children may be less exposed to and dependent on family dynamics as they have more opportunity to seek support elsewhere if they experience difficulties at home. This is in line with the idea of a gradual shift in focus from the family to friends [34]. When we in the present study actually control for children’s psychological vulnerabilities, it is possible that the effects are exacerbated, because for older children the support from friends is even more important and at the same time, the everyday changes restricted physical contact with friends.

## Strengths and limitations

A clear strength of the present study, is the use of longitudinal data that allowed us to control for children’s psychological vulnerability (i.e., symptoms of depression and anxiety assessed prior to the pandemic). In contrast to previous cross-sectional studies, we have been able to rule out confounding effects of children’s general adjustment. Another strength is the focus on children’s reactions more broadly with the opportunity to examine the normal range of reactions, including positive and negative reactions. However, the study also has some limitations that deserve mentioning. First, to assess children’s reactions, we asked children to rate their reactions in comparison to before the pandemic. We acknowledge that the reliability of retrospective questionnaires may be subject to memory or recall bias, particularly when assessing personal life events. However, we assessed children’s reactions, which might be less subject to bias and being able to assess a change in children’s reactions outweigh the probability of any measurement error. Second, our measure of screen time use included both social media use and online gaming. We may have missed an opportunity to see a relationship with children’s reactions, as we did not assess these two types of screen time separately. We, therefore, encourage future studies to use more nuanced measures that, for example, probe social media use and online gaming separately. It would also be interesting to explore how much communication children have with other gamers or friends during online gaming sessions. We speculate that children may use gaming as a way of connecting and communicating with friends, and thus, gaming might have protective mechanisms on children’s reactions [53]. Third, we have eluded to the fact that children may have met their friends physically despite the public health recommendations, something we did not ask children about. Finally, we urge caution in generalizing the results to the general population, as our sample may be characterised as a non-representative convenience sample and further, data for the present study were drawn from the XXX study, where families were recruited when parents attended family welfare centres for mediation, counselling or family therapy (i.e., vulnerable families).

## Conclusion

In summary, the present study found both positive and negative consequences of the pandemic on children; children reported fewer emotional reactions and more somatic/cognitive reactions. We observed associations between most of the COVID-19 related predictors and children’s reactions, and further, age moderated several of these associations; older children were more severely impacted by the changes than were younger children. This fits with a developmental perspective of adolescence as a time of heightened emotional sensitivity and developing autonomy. Going forward, it will be important to replicate or even extend the present study by focusing on an even broader spectrum of child reactions to the pandemic, and to explore what the impact of school re-opening (and closing and re-opening again) has on children as the pandemic continues on.
